# Calciphylaxis in chronic renal failure: An approach to risk factors

**DOI:** 10.4103/0971-4065.57109

**Published:** 2009-07

**Authors:** W. Rezaie, H. A. J. M. Overtoom, M. Flens, R. J. L. Klaassen

**Affiliations:** Department of Internal Medicine, Zaans Medical Center, The Netherlands; 1 Department of Radiology, Zaans Medical Center, The Netherlands; 2 Department of Pathology, Zaans Medical Center, The Netherlands

**Keywords:** Calciphylaxis, chronic renal failure, panniculitis, secondary hyperparathyroidism

## Abstract

We present a case of calcifying panniculitis due to calciphylaxis in a nontherapy compliance 65-year-old man suffering from chronic renal failure. Calciphylaxis, a life threatening condition, is characterized by high calcium × phosphate product, presence of calcium crystals in the skin and secondary hyperparathyroidism. The clinical presentation includes painful firm plaques, which could progress to nonhealing ulcers. A review of literature is given with emphasis on identification of risk factors and early diagnosis.

## Introduction

Patients with an edematous painful leg are frequently referred to internists. Differential diagnosis includes a wide range of diseases, such as deep venous thrombosis and infections. Herein, we report a patient with chronic renal failure (CRF) suffering from bilateral painful warm, swollen legs that appeared to be caused by an insufficient dialysis dose, and its early diagnosis and timely treatment prevented serious complications. Although this condition is known to nephrologists, it remains under recognized by other physicians including internists and general practitioners.

## Case Report

A 65-year-old Indonesian man was referred to our clinic because of progressively painful plaques in the skin of his legs during a 3-week period. The patient had been diagnosed with diabetes mellitus type II at age 38 and hypertension at age 52. He had developed chronic renal failure and had begun maintenance continuous ambulatory peritoneal dialysis (CAPD) at the age of 62. Peritoneal dialysis was hampered by a poor adherence to the prescribed treatment protocol, a poor compliance to the prescribed medication and by an excessive oral fluid intake of about 5 L/day to which he had been used to since his early youth in a tropical country. Other medical history included myocardial infarction and an ischemic cerebrovascular incident at the age of 62. His medication consisted of acetylsalicylic acid 100 mg daily, diltiazem 180 mg daily, darbepoetin adapted to heamoglobin level, Insulin adapted to blood glucose level, calcium-acetate 1 g daily, sevelamer 2.4 g daily, alfacalcidol 1 microg daily, simvastatine 40 mg daily, and bumetanide 1 mg daily.

**Figure 1:**
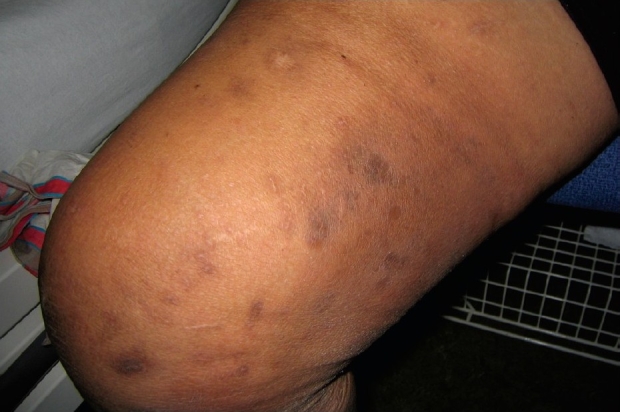
Multiple painful indurated plaques on both lower extremities

On physical examination, the patient appeared to be in pain, had low grade fever (38.4°C) with normal vital signs. There were diffuse, conflating painful, firm, indurated plaques consistent with infiltrates on both thighs and lower legs [Figure 1], where previously only a considerable amount of pitting edema had been present. Some darker spotty discoloration in the infiltrates suggesting local necrosis was noted. Peripheral pulses were weakly present bilaterally. The patient had a BMI of 39 kg/m^2^. Further examination disclosed no other abnormalities.

Laboratory studies are showing Table 1. Microbiological studies (cultures, serology) disclosed no abnormalties.

**Table 1:** Results of laboratory study

Variable	Outcome	Normal range
Potassium (meg/L)	4.9	3.6–4.8
Sodium (meg/L)	128	134–146
Creatinine (mg/dL)	18.5	64–108
Urea (mmol/L)	39.2	3.0–7.5
Alkaline (phosphate u/L)	1	40–120
Albumine (g/L)	31	35–50
Calcium (mmol/L)	2.22	2.15–2.68
Phosphate (mmol/L)	3.26	0.8–1.40
Parathormone (pmol/L)	134.10	1.60–6.90
C reactive proteine mg/L	246	<10
White cell count (× 10^9^/L)	11.0	4.5–10
Hemoglobin (g/dL)	5.9	8.9–10.7
Hematocrit (%)	30	41–50
Platelets (× 10^9^/L)	287	150–40

X-ray studies of the lower legs revealed soft-tissue calcifications in the regions, found to be indurated at physical examination [Figure 2a]. Duplex ultrasound studies of arteria femoralis, arteria tibialis posterior and arteria dorsalis pedis did not show any significant obstruction. Computed tomography (CT) scanning of the patient's lower legs showed calcification of vessels, mild atrophy of different muscles and diffuse subcutaneous calcification [Figure 2b].

**Figure 2a:**
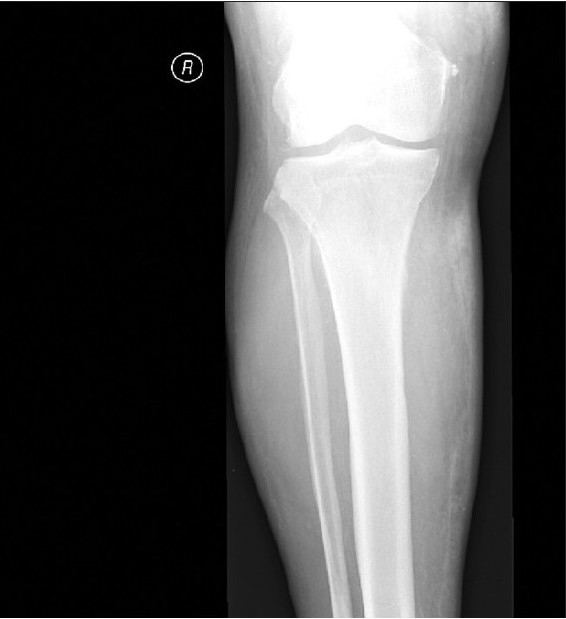
Soft tissue x-ray showed increased density on medial aspect of right lower leg

**Figure 2b:**
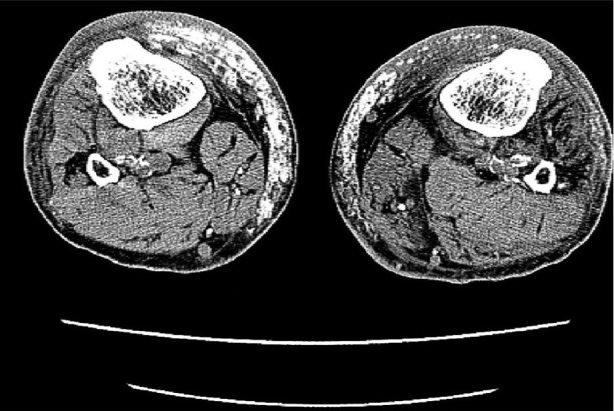
CT images of lower extremities showed calcified vessels and subcutaneous calcification

Skin biopsy specimens were taken from the plaques demonstrating calcium depositions involving the intima of small arteries and venules. The specimens reveals extravascular soft tissues calcifications, associated with lobular panniculitis and adipose tissue necrosis [Figure 3a]. The presence of the calcium-salt-depositions was confirmed by a specific calcium-staining (von Kossa stain) [Figure 3b]. On basis of this findings and elevated serum Ca × P product, the diagnosis ‘calciphylaxis’ was made. The first step of therapy was normalization of Ca × P product serum level. For this reason, calcitrol and calcium acetate were discontinued and low calcium dialysate (calcium concentration 1.25 mEq/L) was instituted. Furthermore, the dialysis-dose was increased (from 8 l dialysate/day to 12 l/day). Within a few days, the results of the laboratory findings greatly improved and within a week, the legs became less inflamed and painful and patient became ambulatory. In that week, corticosteroid-therapy was considered to decrease the inflammatory response but not instituted because of lack of scientific evidence for this measure.

**Figure 3a:**
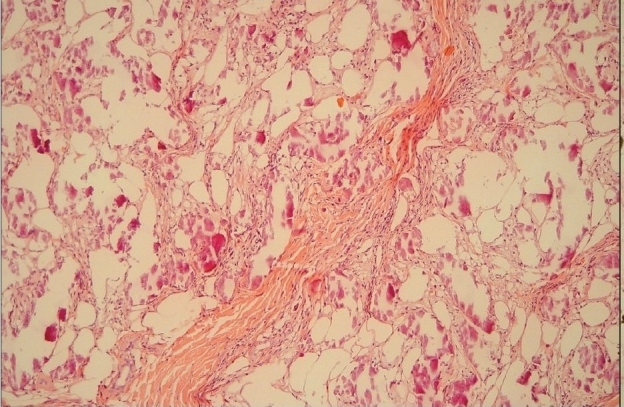
H and E stain of skin lesions showed lobular dystrophic calcified panniculitis and adipose tissue necrosis

**Figure 3b:**
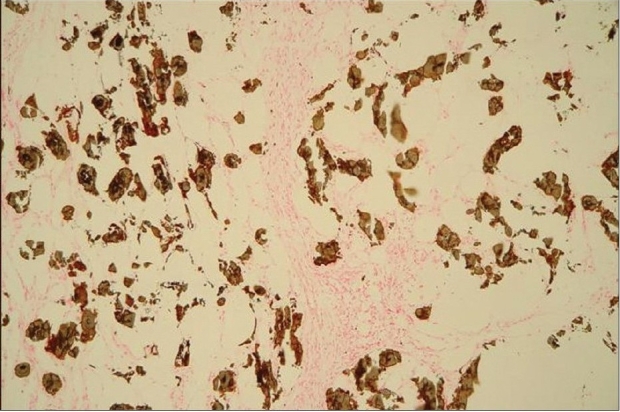
A von Kossa stain of the same case demonstrates calcium deposits in subcutaneous fat and in the walls of vessels

## Discussion

Calciphylaxis (or calcific uremic arteriolopathy) is a serious life-threatening condition characterized by progressive calcium salt deposition in small and medium sized vessel and superficial soft tissue.[1–3] It is estimated to occur in 1–4% of CRF-patient who are subjected to hemodialysis each year.[4]

The clinical presentation includes primary lesions as painful firm plaques or subcutaneous nodules which progress to secondary lesions as non-healing ulcers.

This process can result in tissue necrosis, gangrene and sepsis.[3,5] The lesion manifests itself characteristically in lower extremities particularly the thighs and buttocks, but less frequently in abdomen, upper extremities and penis.[1]

Calciphylaxis was first described by Selye, in 1962 as a local or systemic calcium deposition in an experimental animal model. He postulated that deposition are induced by exposure to the “challenging” agents such as local traumas or metal salt after a phase of developing systemic “sensitization” to one or several agents such as parathyroid hormone, vitamin D or a diet high in calcium and phosphate.[6,7] Although the pathogenesis of calciphylaxis is poorly understood, Liach *et al*. outlines three pathogenic factors presence of uremic milieu in concert with a high Ca × P product; elevated content of calcium salt in the skin and presence of high parathyroid hormone (PTH) level.[8] Lab studies of our case confirm contribution of above mentioned parameters to develop calciphylaxis. [Table 1] Several risk factors in CRF patient appear to be associated with increase of Ca × P product level which is shown in Table 2. Obesity, hypoalbuminemia, decreased local or systemic blood flow, pre-existing disturbance of coagulation and trauma are reported to be predisposing factors in developing secondary lesions.[1,4,9,10]

**Table 2:** Risk factors for calciphylaxis

High intake of calcium and phosphate
Use of calcium-containing phosphate binders
Use of calcitrol
Inadequate dialysis dose
Use of dialysate with high calcium concentration
Secondary hyperparathyroidism
Obesity
Hypoalbuminemia

Secondary hyperparathyroidism, an important risk factor is present commonly in the setting of CRF. Recent studies revealed that this condition develops in response to hyperphosphatemia, low calcium level and deficit of calcitrol.[8] Hyperparathyroidism is a serious problem in dialysis patient which accelerates bone turnover and consequently phosphate and calcium efflux.[11] Hence, early control of secondary hyperparathyroidism is essential. Calcitrol administration was considered in the past as an effective treatment for the treatment of secondary hyperparathyroidism, but this implies two relevant side effects including hypercalcemia and hyperphosphatemia.[12]

Therefore, administration of calcitrol was stopped in our case. Over the past years are several vitamin D analogs developed which can inhibit PTH secretion without promoting of hyperphosphatemia and hypercalcemia. Clinical trials to determine long term results of using these agents in dialysis patient are still in progress.[13] Recent randomized clinical trials has shown that cinacalcet, a calcimimetic, effectives decrease serum parathyroid hormone in dialysis patients with secondary hyperparathyroidism.[14,15] Cinacalcet increase the extracellular calcium sensitivity of the calcium receptor on the parathyroid cells by lowering the threshold for activation by calcium ions.[14–16]

Parathyroidectomy in patient with high PTH level appears to ameliorate clinical presentation, but there is no strong evidence regarding improved survival.[4]

In addition, Block *et al*. showed that hyperphosphatemia occurs in 50% of dialysis population.[13] The most common causes of hyperphosphatemia consist of high dietary intake of phosphate and inadequate dialysis.[17] With non compliance as in our case, hyperphosphatemia may be a reflection of insufficient dialysis.[12] This necessitated application of more intensive dialysis program with low calcium dialysate (0.00 to 2.00 mEq/L). Furthermore, restriction of oral intake of calcium and phosphate was considered. The use of non calcium phosphate binders such as Sevelamer are advocated in such patient. A recent study showed that use of sevelamer in CRF patient is associated with significant survival benefits compared to calcium containing phosphate binders.[13] Inadequately dose of sevelamer before admission of patient in our clinic contributed to the high serum phosphate. This reason increase dose in the sevelamer to 4.8 gm/day.

Multiple cases are described in literature with improvement of calciphylaxis symptoms after application of sodium thiosulphate, prednisone and hyperbaric oxygen in conditions unresponsive to the above outlined treatment.[18–20]

Though after application of several measures, the clinical presentation of our patient was significantly improved, the long-term prognosis in such patient remains poor. Fine *et al*. demonstrated a mortality rate of 33% at six months in patient who presented only plaques which increased to above 80% in those patients developing ulceration.[3]

## Conclusion

With the increasing number of patients undergoing dialysis, calciphylaxis is no longer rare. It is important for the clinician to be aware of this entity, because prompt diagnosis and treatment may halt progression of disease and minimize mortality rate. Avoidance or correction of risk factors described in this study is the first step to prevent calciphylaxis in CRF patients.
